# Reduced Expression of Prion Protein With Increased Interferon-β Fail to Limit Creutzfeldt-Jakob Disease Agent Replication in Differentiating Neuronal Cells

**DOI:** 10.3389/fphys.2022.837662

**Published:** 2022-02-18

**Authors:** Gerard Aguilar, Nathan Pagano, Laura Manuelidis

**Affiliations:** Section of Neuropathology, Department of Surgery, Yale Medical School, New Haven, CT, United States

**Keywords:** innate immunity, microglia, Transmissible Spongiform Encephalopathies, SV40 T antigen, infectivity, viral DNA

## Abstract

Immortalized uninfected septal (SEP) neurons proliferate but after physiological mitotic arrest they express differentiated neuronal characteristics including enhanced cell-to-cell membrane contacts and ≥ 8 fold increases in host prion protein (PrP). We compared proliferating uninfected and Creutzfeldt-Jakob Disease (CJD) agent infected cells with their arrested counterparts over 33 days by quantitative mRNA and protein blot analyses. Surprisingly, uninfected arrested cells increased interferon-β (IFN-β) mRNA by 2.5–8 fold; IFN-β mRNA elevations were not previously associated with neuronal differentiation. SEP cells with high CJD infectivity titers produced a much larger 40–68-fold increase in IFN-β mRNA, a classic host anti-viral response that is virucidal for RNA but not DNA viruses. High titers of CJD agent also induced dramatic decreases in host PrP, a protein needed for productive agent replication. Uninfected arrested cells produced large sustained 20–30-fold increases in PrP mRNA and protein, whereas CJD arrested cells showed only transient small 5-fold increases in PrP. A > 10-fold increase in infectivity, but not PrP misfolding, induced host PrP reductions that can limit CJD agent replication. In contrast to neuronal lineage cells, functionally distinct migratory microglia with high titers of CJD agent do not induce an IFN-β mRNA response. Because they have 1/50th of PrP of an average brain cell, microglia would be unable to produce the many new infectious particles needed to induce a large IFN-β response by host cells. Instead, microglia and related cells can be persistent reservoirs of infection and spread. Phase separations of agent-associated molecules in neurons, microglia and other cell types can yield new insights into the molecular structure, persistent, and evasive behavior of CJD-type agents.

## Introduction

Host prion protein (PrP) mRNA transcription sharply increases during brain synapse formation (Lieberburg, 1987; Manson et al., 1992). In neuronal lineage cells PrP localizes ultrastructurally on the plasma membrane and rough endoplasmic reticulum (Manuelidis, 2007). Host PrP is needed for the replication of the infectious agents that cause Transmissible Spongiform Encephalopathies (TSEs) as shown by PrP knockout studies in scrapie (Büeler et al., 1993). Conversely, transgenic PrP overexpression increases susceptibility to infection and reduces the incubation time to terminal disease in mice (e.g., Manuelidis et al., 2009b). It is now commonly believed that the causal infectious agent in TSEs is a misfolded form of host PrP without any nucleic acid component (Prusiner, 1998). However, claims that misfolded PrP itself can become infectious have not been reproducible (Timmes et al., 2013; Barron et al., 2016). Furthermore, all highly infectious CJD and scrapie preparations contain significant amounts of long nucleic acids when evaluated using contemporary detection techniques (Manuelidis, 2011). Moreover, nucleases that destroy nucleic acids but have no effect on PrP, reduce infectivity by ~1,000 fold (Botsios and Manuelidis, 2016). Misfolded PrP forms amyloid fibrils and on Western blots displays truncated proteinase K resistant bands (PrP-res) and nucleic acids, including DNA aptamers, can promote PrP fibrillation as shown in phase separation studies (Matos et al., 2020; do Amaral and Cordeiro, 2021). While cellular PrP-res is used for diagnosing mammalian TSE agent infections, PrP-res can represent pathological fibrillar products of old agent interactions. In fact, PrP-res continues to accumulate even after infected cells have lost all of their infectivity (Miyazawa et al., 2012).

From a viral perspective: (1) host PrP can act as a membrane receptor and/or intracellular scaffold for agent entry and replication; when infectious TSE agents attach to PrP they may induce PrP misfolding and amyloid aggregation (Manuelidis, 2013). Virus-host protein interactions are reported to induce host protein aggregation into a “prion-like” structure that stimulates host innate immune interferon type I responses (Hou et al., 2011). (2) An agent nucleic acid component can also account for the diversity, uniqueness, stability and virulence of different TSE agents (Manuelidis, 2010). Each TSE strain displays a distinct doubling time of 7–33 days, in addition to its own region-specific brain pathology in non-transgenic mice (Miyazawa et al., 2011). Moreover, most agents induce the same PrP-res brain bands in these mice (Manuelidis et al., 2009a). Conversely, PrP-res band patterns can be altered by propagation in neural cell lines yet this has no biological effect on agent characteristics when cells are later inoculated in mice (Arjona et al., 2004). A TSE strain, like many viral strains, can prevent superinfection by a different strain (Manuelidis and Lu, 2003; Nishida et al., 2005). (3) While the molecular structure of TSE agents remains unknown, virus-like dense particles of 20–25 nm within infected CJD and scrapie cells (Manuelidis et al., 2007) are also concentrated in highly infectious preparations with reduced or no PrP/PrP-res (Manuelidis, 2007; Kipkorir et al., 2014). These particles, unlike PrP amyloid fibrils, do not bind PrP antibodies (Manuelidis, 2007; Manuelidis et al., 2007). Finally, (4) the vast majority of TSE infections can be traced to an external source and environmental controls can prevent infection, e.g., the disappearance of human kuru after the cessation of ritual cannibalism (Gajdusek, 1977) and the sharp decline of epidemic “mad cow disease” (BSE) after contaminated feed was banned. Even widespread endemic sheep scrapie infections have been controlled by rigorous environmental disinfection with no spontaneous reappearance (Sigurdarson, 1991). Environmental TSE agents would be expected to induce interferon-β (IFN-β), a classic host innate immune response to foreign pathogens that creates an antiviral state (Nagarajan, 2011; An et al., 2019). We here show that the infectious agent but not PrP/PrP-res induces IFN-β mRNA.

Late-stage neuronal degeneration in TSEs has received much attention, but tissues and cells responsible for the initial agent entry, dissemination, and asymptomatic persistence are less studied. Bone marrow derived cells and tissues, not neurons, are the first stop for TSE agents in peripheral infections including the oral route (Shlomchik et al., 2001). Scrapie homogenates inoculated intramuscularly induce significant agent titers in spleen 8 weeks before infectivity is detectable in the brain (Eklund et al., 1967) although serum and plasma were not infectious. Subsequent CJD studies showed white blood cells (WBCs) carried the infectious agent (Manuelidis et al., 1978, 1985) even though these cells have very low or no PrP (Choi et al., 2009). Many different viruses (Nikitina et al., 2018), including “neurotropic” poliovirus and SARS-Cov-2 (Pagano et al., 2020) as well as human papova viruses (An et al., 2019), are carried by WBC to spleen and lymph nodes before they gain a foothold in brain, and WBC can carry other TSE agents (Douet et al., 2016). Myeloid lineage macrophage/dendritic cells account for only 1–2% of the WBC population but can carry TSE agents to the gut (Radebold et al., 2001; Shlomchik et al., 2001). After intraperitoneal injection, scattered long-lived myeloid/dendritic cells in the submucosal intestinal lymph nodes show *de novo* misfolded PrP indicative of infection at 4 weeks whereas this change first appears in brain at 25 weeks. This gut myeloid/dendritic cell infection persists until terminal illness (Radebold et al., 2001). Infectivity titers in scrapie also show myeloid/dendritic type cells in spleen are chronic reservoirs during protracted asymptomatic infections with a constant plateau of infectivity throughout life (Eklund et al., 1967). And, humans inoculated peripherally with CJD contaminated growth hormone have developed neurological symptoms 38 years after exposure (Croes et al., 2002). Indeed, misfolded PrP in lymphoid tissue biopsies have been used to estimate TSE infections in asymptomatic populations (Hilton et al., 2004). Microglia in brain, another long-lived myeloid dendritic cell type, also contributes to an antiviral response. Microglia are cellular and morphological chameleons that can act as Trojan horses for brain infection (Manuelidis et al., 1997), and these cells classically participate in lytic responses and antigen presentation. Long lived and migratory activated microglia, with 1/50th of the PrP of an average brain cell and no detectable PrP-res, have CJD agent titers as high as total brain (Baker et al., 2002) yet their responses to infection are different from those seen here in SEP neuronal cells.

Interferon and PrP changes during physiologically induced differentiation in uninfected cultured rat neuronal septal (SEP) cells were first detailed. These responses were then compared to those seen during FU-CJD infection by both RT/qPCR for mRNAs and parallel protein blot assays during several weeks of arrest. Post-mitotic septal neurons immortalized with an SV40 T antigen (Tag; Eves et al., 1994), previously showed that a shift from 33°C to 37.5°C in reduced serum was sufficient to arrest proliferation induced by the Tag construct (Miyazawa et al., 2010). A more differentiated SEP cell morphology with specialized cell-to-cell contacts, nanotube formation, and an 8-fold increase in PrP was seen by 1 week of arrest and PrP increases were magnitudes higher than those seen in other PrP models (e.g., Castle and Gill, 2017). Moreover, SEP cells infected with the FU-CJD agent also produced >10-fold more infectious particles after arrest (Miyazawa et al., 2012). Changes in IFN-β, Tag, PrP and infectivity titers per cell are detailed here. Surprisingly, host PrP synthesis was significantly suppressed by infection as compared to uninfected controls. Additionally, infection induced a sustained 40–68-fold increase in IFN-β mRNA, a futile host response to control increasing titers of the FU-CJD agent.

## Materials and Methods

### Conditions for Proliferation and Arrest of Normal (NL) and FU-CJD Infected SEP Cells

The SV40 T antigen (Tag) stimulates cell proliferation, and a temperature sensitive Tag was used to immortalize post mitotic septal neurons in culture. Low Passage SEP cells (AS583 subclone e422) without detectable astrocytic GFAP were a generous gift of Wainer (Eves et al., 1994). These low passage proliferating cells were frozen at p3 (designated “p1” here) and maintained in high glucose DMEM with 10% FBS, penicillin/streptomycin (50 units/ml), and G418 (0.2 mg/ml) at the permissive temperature of 33°C and were split 1:4 every 4 days per passage. Two low passage SEP cells lines infected with the FU-CJD agent (Miyazawa et al., 2012) were frozen at passage 17; the first line, infected during proliferation, was maintained in a proliferating state in 10% FBS/33°C. These cells had a low titer of 0.4TCID per cell. They were then arrested (“FU-CJD arrested”). The second FU-CJD infected line had been maintained under chronic arrest conditions (2% FBS/37.5°C) and when frozen had a high titer of 5 TCID/cell (Miyazawa et al., 2012) and designated “FU-CJD re-arrested” cells. Both cell lines were thawed at passage 17 and split 1:4 every 4 days for three passages in 10% FBS at 33°C to increase cell numbers needed for multiple sequential protein and RNA sequential studies. For arrest, uninfected- and FU-CJD infected SEP cells at ~60% confluence were shifted to 37.5°C in 2% FBS DMEM and re-fed every 2 days with 2% FBS DMEM as previously described (Miyazawa et al., 2012); ~4% of arrested cells showed DNA proliferation by nuclear BrdU incorporation at later times allowing cells to be split 1:2 from 14 to 33 days of arrest. For protein analyses and RNA extractions, cells were collected from 6 to 33 days after arrest (*t* = 0). Four sets of proliferating SEP cells were tested for arrest: starting p7 (28 days), p21 (84 days), p25 (100 days), and p41 (164 days). Multiple flasks of proliferating and arrested cells were carried in parallel for each set and tested at sequential time points; some arrested cells were returned to a proliferative state after an extended arrest to document a *t* = 0 days state.

### Western Blot Analysis

Cells dislodged from flasks in PBS with 0.6 mM PMSF were centrifuged at 1,500 *g* for 10 min, suspended in 50 mM Tris–HCl pH 8.9 and sheared by bath sonication. All antibodies were from Santa Cruz except β-actin (Sigma) at the following concentrations: PrP (clone 6D11) @1:15,000, SV40 Tag (Pab 419) @1:500, α-tubulin (clone A-6) @1:750, β-actin (clone AC-15) @1:6,000 and Annexin II (clone C-10) @1:500. All these primary mouse antibodies were detected using m-IgG-kappa BP HRP @1:800 as the secondary antibody. For quantitation, chemiluminescent signals on blots were normalized with respect to protein loaded/lane and additionally confirmed by β-actin, α-tubulin, and Annexin II.

### Infectivity Assays of Re-arrested FU-CJD Cells

As previously detailed (Liu et al., 2008; Miyazawa et al., 2012), the tissue culture infectious dose (TCID) in each SEP cell homogenate, at several dilutions, was assayed by exposing duplicate wells seeded with moue neuronal GT1 indicator cells. Uninfected SEP cells elicited no PrP-res in GT1 cells, as shown in many previous experiments. In contrast, *de novo* PrP-res was produced only after infection. High titer inocula induce PrP-res at early GT1 passages whereas lower titer samples needed more passages to provoke *de novo* PrP-res. Ten-fold dilutions of proliferating and re-arrested FU-CJD infected SEP cells previously documented quantitative induction of PrP-res (Miyazawa et al., 2010). FU-CJD infected GT1 cells passaged for >1 year reproduced the original clinical signs and brain lesion profiles as displayed by the FU-CJD strain in brain (Arjona et al., 2004).

### RNA Extraction and RT/qPCR for Quantitative mRNA Analysis

RNA was isolated from pelleted SEP cells following the NEB Monarch Total RNA Miniprep Kit instructions, including the 15 min DNAse incubation step. Kit columns were loaded with E7-2.5xE7 cells yielding a total of 0.5–2μg RNA, a value below the maximal 100 μg column capacity. RNA in nuclease-free water was quality checked and quantified by Nanodrop spectrophotometry using OD 260/280 and 260/230 ratios of ~2 as indicators of high quality RNA. Because full length synthetic RNAs targets were not available, Glyceraldehyde-3-phosphate dehydrogenase (GAPDH) dilution curves were performed to quantitate and normalize each RNA sample (see Supplementary Figures 1 and 2). RT/qPCR input dilutions for each primer pair were determined by a standard curve of three serial dilutions run in duplicate or triplicate in the linear range to find the optimal RNA load giving a Cq value on the standard curve. Each assay included duplicate no-template (H_2_O) negative control. The numerical mean and SEM of a minimum of six determinations in three or more separate assays were done for each sample.

RT/qPCR NEB reactions (14 ul total volume) contained 2x Master mix (7 μl for 1x final concentration), 20x RT enzyme (0.7 μl), 5 μM Forward and Reverse primers (0.84 μl for 300 nM each), nuclease-free water (3.46 μl), and RNA template (2 μl). GAPDH was used as an internal control to normalize the relative quantity of RNA in each sample and time point. Most RNAs could be quantitated with an input of 100 pg. Neurofilament protein-M (NFP) and interferon (IFN) mRNAs required higher inputs of 30–200 ng of RNA. Reactions were run on a MyGo Mini real-time thermal cycler as previously described (Pagano et al., 2020). The thermal profile for the following primers consisted of one RT step at 55°C for 10 min followed by one denaturation at 95°C for 60s, and 40 cycles at 95°C for 10s, 10s at T anneal temperatures (Table 1), and extensions at 72°C for 20s. Table 1 lists the primers, annealing temperatures and products for each primer pair. Each run was followed by melting curve analysis for Tm of products. None of the primer pairs generated spurious extra peaks, e.g., see Supplementary Figure 3.

**Table 1:** Primer sequences and annealing temperatures used (T-anneal) with the Tm and Product bp length.

Gene	Primer sequences	Product Tm	T-anneal	Product (bp)
GAPDH	F: 5’-CAACTCCCTCAAGATTGTCAGCAA	85.75°C	54°C	118
	R: 5’-GGCATGGACTGTGGTCATGA			
Actin	F: 5’-AAGTCCCTCACCCTCCCAAAAG	86°C	55°C	97
	R: 5’-AAGCAATGCTGTCACCTTCCC			
SV40-T	F: 5’-GATGCAACTGAGATTCCAACCT	82°C	53°C	192
	R: 5’-GCAATTCTGAAGGAAAGTCC			
PrP	F: 5’-CGTCACCCAGTATCAGAAGGAG	86°C	57°C	308
	R: 5’-CTGAAGCGAATAGCATCTGGTC			
NFP-M	F: 5’ATCACTTGGAGGAAGACATCCACCGG	90.6°C	62°C	864
	R: 5’-TTCCTCTGCAATGACTGTAGGGC			
IFN-β	F: 5’-CGTTCCTGCTGTGCTTCTC	86.2°C	53°C	150
	R: 5’-TGTAACTCTTCTCCATCTGTGA			
OAS 2	F: 5′- CCTATGATGCACTAGGTCAGCTGC	88°C	69°C	470
	R: 5′- TAGAAGATGCCAACACCAGCGGTC			

*Each Tm refers to the final RT/qPCR product’s melting temperature and determined empirically by melting curve calculations of results. Primer pair references: GAPDH (Schirmer et al., 2019), Actin (Sun et al., 2020), SV40 Tag (Wolfe et al., 2008), NFP (Uchida et al., 2004), IFN-β (Adriaansen et al., 2007), and OAS2 (Perelygin et al., 2006). Rat PrP primers were designed by John Davis*.

## Results

**Figure 1:**
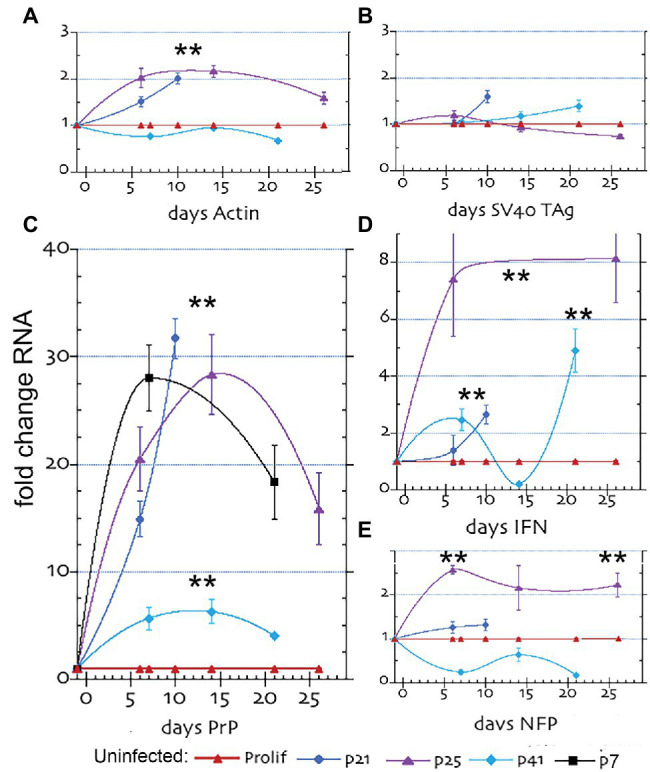
Quantitative RT/PCR (RT-qPCR) results for mRNA fold changes in uninfected SEP RNAs. The graphs show the sequential fold change of RNA expression with arrest after different passages; where p7 is shown in black, p21 in blue, p25 in purple and p41 in turquoise with parallel proliferating non-arrested controls in red. **(A)** β-actin, **(B)** SV40 Tag, **(C)** PrP, **(D)** Interferon β (IFN) and **(E)** Neurofilament protein-M (NFP). mRNA levels show the mean and SEM of a minimum of 6 determinations for ≥3 separate RNA aliquot assays. The ** in all plot figures indicates a *p* ≤ 0.002 by student’s unpaired *T*-test.

Uninfected immortalized SEP cells were not previously evaluated for more than 8 days after proliferative arrest. We first extended these times to define induced transient vs. sustained changes. Physiological induction into an arrested non-proliferating state using a 4.5°C temperature shift to 37.5°C in low serum was capable of arresting cells for 25–33 days. Previously, neuronal differentiation features were enhanced by an 8-day arrest, including 8-fold elevations in host prion protein (PrP), an ultrastructural increase in cell-to-cell plasma membrane attachments and formation of many nanotubes from cell to cell (Miyazawa et al., 2010); Neurofilament RNA by Northern blot hybridization has also been reported (Eves et al., 1994). These findings are consistent with many other studies indicating a role for PrP in synaptic plasticity, cell adhesion, and cell signaling, but a central defined function for PrP remains unresolved (e.g., Linden, 2017). The extended studies here, using RT/qPCR for mRNA transcripts, revealed unexpected changes in interferon-β (IFN-β) in uninfected arrested SEP cells. In the following studies, we first assessed the responsiveness and reproducibility of changes induced by arrest in uninfected cells at early to late passages where each passage covers 4 days prior to arrest, i.e., p7 cells were arrested after 28 days of proliferation, p25 after 100 days, and p41arrested after 164 days. All uninfected cell mRNAs are shown in Figure 1. FU-CJD infected cells, arrested at p17 are then compared as later detailed.

### Dramatic Increases in Specific mRNAs in Uninfected SEP Neurons After Arrest

Figure 1 shows the increase in specific mRNAs in uninfected SEP cells, where proliferating control cells for each different passage series were run in parallel and plotted as a 1x control for comparison (red line). In each passage set the proliferating control was comparable. Panel **1A** shows actin increased and remained elevated ~2 fold from 5 to 25 days in cells arrested at p21 and p25 days (blue and purple lines respectively). This small significant change (**p ≤ 0.002) is biologically meaningful because the previous studies demonstrated ~20% of SEP cells became very large and were filled with actin filaments by 8 days after arrest. In contrast, SEP cells arrested at later passage p41 (turquoise line), did not show an increase in actin, suggesting genetic drift by this passage. SV40 Tag mRNA (Panel **1B**) showed no meaningful increase at any post-arrest time in each of the three passage series, a finding that would be expected from healthy differentiating neurons that are arrested. Since all SEP cells contain integrated recombinant SV40 Tag used for immortalization, a low level Tag RNA was produced by all cells. This constant 1x level of Tag RNA that was not mirrored by Tag protein levels (see below), i.e., quantitative RNA amounts can be separated from observed protein synthesis. Tag quantitative RNA fidelity was verified by RT/qPCR tests of HEK human cells with and without a Tag construct; Tag negative cells yielded no positive RNA signal whereas Tag + cells were clearly positive (Supplementary Figure 4).

After arrest, there was an enormous increase in PrP RNA (Figure 1, panel **C**). PrP RNA shot up 16–30 fold after 5 days in p21 (blue) and p25 (purple) arrested cells and was still maintained at 15x proliferating cell levels at 25 days (purple line). In contrast, p41 arrested cells (turquoise line) showed only a 5-fold increase in PrP RNA over 20 days of sampling. This indicated PrP transcription could be compromised by genetic drift in cells passaged for >150 days. To validate if this loss of PrP RNA responsiveness was caused by extended passaging, we investigated a replicate set of early passaged SEP cells arrested at p7. PrP RNA of p7 cells (Panel **C**, black line) were followed for 21 days of arrest and confirmed both the huge increase and maintenance of high PrP RNA levels comparable to those seen in p25 cells passaged for 100 days before arrest.

Interferon-β RNA (IFN-β), shown in panel **D**, was also elevated from 2.7–8 fold over 25 sampling days but showed a more complex response pattern than the other RNA probes. Whereas arrested p25 cells (purple line) maintained a consistent 8-fold increase in IFN-β vs. control proliferating cells (red line), the more extensively passaged p41 cells were variable (turquoise) and showed a significant 2.5-fold and 5-fold increase only at 7 and 20 days, respectively. The 8-fold sustained increases in p25 cells raise the unexpected possibility that IFN-β RNA might increase in neuronal cells during differentiation. The clear increases in IFN-β in uninfected neuronal cells at p25 occurred without a corresponding increase in Tag that can stimulate host IFN-β regulatory factors. The initial and small physiological shift used for arrest SEP cells would be unlikely to produce the sustained 8-fold increase in IFN-β mRNA. Neurons can produce IFN-β as part of an antiviral response (e.g., Delhaye et al., 2006; Paul et al., 2007) but there is a paucity of data on IFN-β transcription in uninfected neuronal cells *in vitro* or *in vivo*. Finally, the neuron-specific neurofilament-M (NFP) marker showed a positive and significant rise after arrest (panel **E**), indicating SEP cells deafferented with neuronal characteristics.

### Quantitative PrP and Tag Protein Analyses in Uninfected SEP Cells

**Figure 2:**
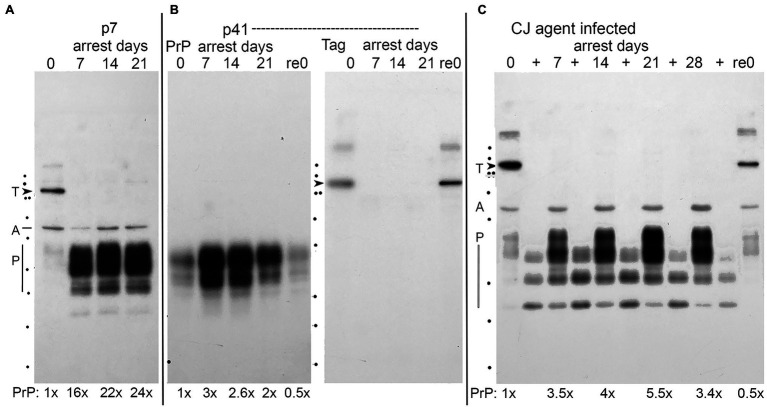
Representative western blots of uninfected (panels **A** and **B**), whereas panel **(C)** shows FU-CJD infected proliferating SEP cells. Day 0 are proliferating cells and effects of arrest are seen at days 7, 14, 21, and 28. Re0 lanes show cells returned to proliferative conditions after longest arrest time. **(A)** Low passage (p7) uninfected SEP cell lysates all loaded at 3E4 cell equivalents (CE) by standard protein assay show 35–20 kDa bands of normal SEP rat PrP (P), and single bands of Actin (A), and SV40 Tag (T, arrowhead, at 90 kDa). PrP-fold amounts, normalized for actin, are indicated below each lane. **(B)** Uninfected SEP cells arrested after continuous propagation for 41 passages (164 days) mount a reduced PrP arrest response, in accord with reduced PrP mRNA in Figure 1. The p41 arrest lanes show lower PrP amounts with the same CE/lane also verified by gold stain total protein. Actin detection was done on the replicate half blot in panel **(B)** that also shows the Tag band only in proliferating lanes 0 and Re0. **(C)** Proliferating FU-CJD cells contain PrP as well as PrP-res in proteinase K (PK) digested (+) lanes loaded with 2x the PrP load (E5 CE). PrP, Actin, and SV40 Tag bands detected on this same blot show equal actin loads and Tag again in these FU-CJD arrested cells is seen only in proliferating samples (lanes 0 and Re0). FU-CJD arrested cells show less PrP than uninfected arrested cells (panel **A**, p7), e.g., or p21 and p25 uninfected cells (data not shown). The % PrP-res (of total PrP) is highest at day 0 (70%) in FU-CJD arrested cells and steadily decreased (day 7 = 30%, day 14 = 20%, days 21 and 28 = 7%). Previous infectivity assays on these SEP infected proliferating day 0 cells yielded low but positive infectivity (0.4 TCID/cell; Miyazawa et al., 2012). kDa markers on left are 150, 100, 75 (double dots), 50, 37 25, 20, 15, and 10.

Panels **A** and **B** in Figure 2 show prion protein increases after arrest of early p7 cells and late p41 cells, respectively. Actin normalized PrP amounts in p7 cells at 7, 14, and 21 days (16–24 fold) were very high, in accord with the large 30-fold PrP RNA elevations. The same high PrP amounts were seen in p25 arrested cells (data not shown). These protein elevations followed RNA changes by a few days, i.e., the highest level of PrP mRNA was seen at 7 days (Figure 1C, black line) whereas PrP on protein blots peaked at 14–21 days. This lag would be expected to allow for translation of nascent mRNA transcripts. Older p41 cells (panel **B**) also showed reduced PrP as compared to earlier passage cells but again mirrored the comparatively reduced PrP mRNA in p41 cells. Blots A and B also show that Tag, a protein that appears only in proliferating cells and that localizes to the nucleus (Miyazawa et al., 2010) produces a visible band at 90kd only in initial proliferating day 0 and arrest released Re0 cell lanes, but not in any arrested cells (lanes 7–21 days, panels **A** and **B**). Since Tag mRNA remained constant at all-time points, the inhibition of Tag translation or enhanced protein breakdown pathways are likely to be involved in its disappearance with arrest. Reduced Tag protein also shows that the vast majority of these uninfected cells are not proliferating.

In sum, arrest of uninfected neuronal SEP cells arrested before 100 days induced up to 30-fold sustained increases in PrP mRNA and protein. Late passage p41 cells showed a diminished PrP response, implicating extensively passaged SEP cells had an altered phenotype after passaging for >150 days. Nevertheless, all arrested cells showed significant increases in IFN-β; in p25 cells 8-fold increases were sustained from 6 to 26 days, and even p41 cells showed a 5-fold elevation. None of these uninfected cells showed an increase in Tag mRNA. However, Tag protein was produced during proliferation, indicating control at the translational level.

### Proliferating and Arrested FU-CJD Cells Show PrP-res, With Low PrP Induced by Arrest

Three sets of stock FU-CJD infected cells were studied: (1) control p17 proliferating cells that had never been arrested, (2) control cells newly arrested (“FU-CJD arrested”), and (3) high titer FU-CJD p17 that were previously arrested (“FU-CJD re-arrested”). For comparison of total PrP in uninfected and infected cells, both sets were loaded with comparable cell numbers/lane. Figure 2 panel **C** shows that FU-CJD infected proliferating cells on blots have low levels of total PrP at day 0, with increasing PrP after arrest (days 7, 14, 21, and 28 days); low PrP is restored after arrested cells are returned to a proliferative state (re0 lane). The low PrP levels in 0 and re0 lanes is comparable to that seen in uninfected proliferating cells at day 0 (panel **A**). Unlike uninfected cells, the PrP increased only 3.5–5 fold with arrest, significantly less than the 20–30x increase in protein of uninfected p7, 21 and 25 cells (panel **A**). All infected cells also displayed PrP-res bands (+ lanes panel **C**), whereas uninfected cells failed to show PrP-res as previously documented (Miyazawa et al., 2012). Infected cells showed an increase in PrP-res after arrest (+ lanes) but the %PrP-res of total PrP declined steadily with the highest value at day 0 (70%) and was still high (30%). All proliferating FU-CJD cells (panel **C**), as uninfected proliferating cells, also showed positive Tag (T at arrowhead; day 0 and re0 lanes), but Tag was not visible in equally loaded lanes of arrested cells. Actin bands (at A) were used for quantitative normalization.

**Figure 3:**
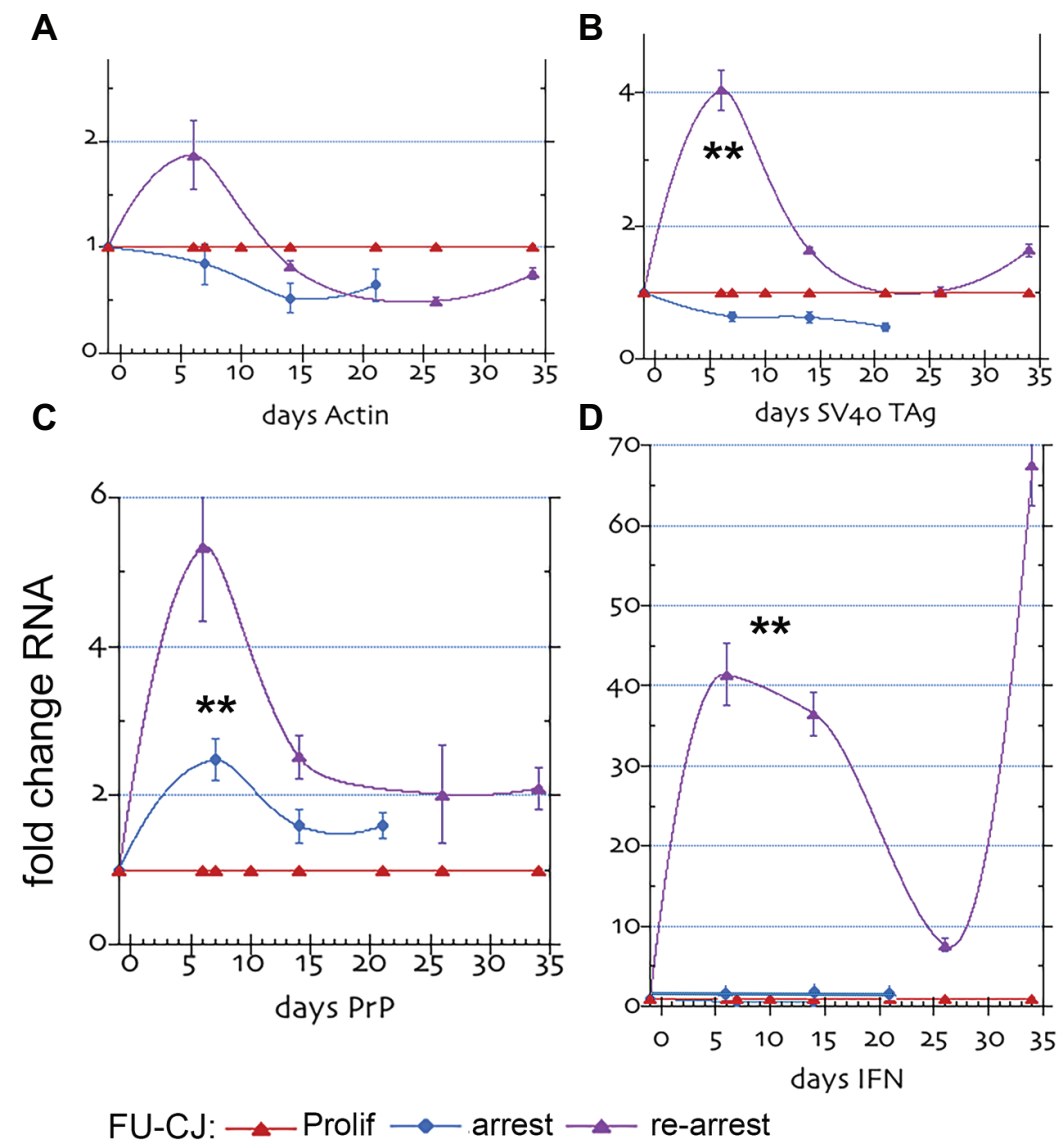
FU-CJD infected SEP mRNAs. The graphs show the fold mRNA change for **(A)** β-actin, **(B)** SV40 Tag, **(C)** PrP and **(D)** Interferon-β in arrested and re-arrested FU-CJD cells as compared to FU-CJD proliferating cells. mRNAs are shown (mean and SEM on same number of samples as in Figure 1). The low IFN-β value at day 26 may show a transient change, or, more likely a non-responding flask.

Quantitative RT/qPCR studies further validated and correlated with the above PrP protein changes in FU-CJD infected cells (Figure 3). In these graphs, proliferating infected cells (red line) are compared with parallel arrested cells at p17 (blue line), and p17 re-arrested cells (purple line). The PrP mRNA was significantly increased (**) in the FU-CJD arrested (Figure 3 panel **C**, blue lines), but only by 2.7 fold, a markedly lower level than the 20–30 fold RNA elevations seen in uninfected cells from p7 to p25 (Figure 1, panel **C**). These low PrP mRNAs levels correspond well with the reduced protein levels. Furthermore, arrested FU-CJD cells did not sustain a significant elevation in PrP mRNA at later time points, unlike uninfected cells. Arrested CJD cells also showed no significant changes in Actin mRNA (panel **A**), SV40 Tag (panel **B**), or IFN-β (panel **D**), and all these panels yielded the same 1x amounts as their proliferating controls (blue and red lines, respectively, in Figure 3).

### mRNAs in Re-arrested FU-CJD Cells Show a Different Pattern of PrP, Tag, and IFN-β

Figure 3 panels show p17 control FU-CJD proliferating cell RNAs in red. These 1x RNA levels were the same as the uninfected controls run in parallel with each other during RT/qPCR; this allowed direct cross comparisons of the various RNAs in uninfected and infected cells. While actin RNA levels were not significantly changed in any FU-CJD infected cells (panel **A**), other markers revealed distinct patterns and quantitative amounts compared to uninfected controls and to each other. PrP mRNA in re-arrested FU-CJD cells (purple line), as in arrested CJD cells, showed a much smaller increase with arrest when compared to uninfected cells. In Figure 3, panel **C**, this increase reached only 5.4x of the non-arrested FU-CJD controls at 6 days and was not sustained, unlike the sustained large increases in uninfected cells that rose to ~30 fold in p7, p21, and p25 cells. These data strongly indicate that FU-CJD agent infection can limit host PrP production, and consequently limit agent replication. The PrP mRNA reductions FU-CJD could involve transcriptional inhibition or enhanced mRNA destruction. These FU-CJD cells were taken at p17, well before there was any reduced PrP responsiveness of SEP cells.

Unlike Tag mRNA in FU-CJD proliferating and arrested cells, re-arrested FU-CJD cells showed a transient small 4-fold increase at 6 days (purple line, panel **B**). The elevated Tag RNA at 6 days in these cells could lead to increased Tag protein synthesis at later arrest days, and additionally influence other transcriptional changes associated with cell proliferation and/or stimulation of known interferon pathways, e.g., STAT/JAK activation.

Most remarkable was the huge increase in IFN-β RNA that far exceeded the Tag mRNA elevation. Figure 3, panel **D**, shows that re-arrested FU-CJD cells displayed enormous and persistent levels of interferon mRNA (40–68-fold) that could not be accounted for by the transient 4x Tag RNA elevation. IFN-β mRNA elevations in re-arrested FU-CJD cells were much higher than the highest 8-fold IFN-β RNA rise obtained in uninfected cells. FU-CJD proliferating and arrested cells showed no significant elevation of IFN-β. To further verify the enormous IFN-β RNA levels detected in early and late re-arrested FU-CJD cells, we evaluated transcription of the interferon-sensitive gene 2′-5’oligoadenylate synthetase (OAS). At 200 ng RNA input, the 0 day proliferating samples repeatedly showed only a questionable positive or no response (Cq of 34–36). In contrast, far fewer PCR cycles gave strong positive signals at 6 days (Cq of 30) and at 33 days (Cq of 26), representing a 7.8-fold relative rise in OAS between 6 and 33 days, verifying IFN-β gene stimulation. Because Tag mRNA was not elevated at 33 days, the 33-day OAS elevation could not be caused by Tag mRNA or protein. To clarify a direct role of the infectious CJD agent, we evaluated both PrP/PrP-res in addition to infectious titers in re-arrested FU-CJD cells at 12 and 33 days.

### PrP Protein Changes and Infectious Titers of Re-arrested FU-CJD Cells

**Figure 4:**
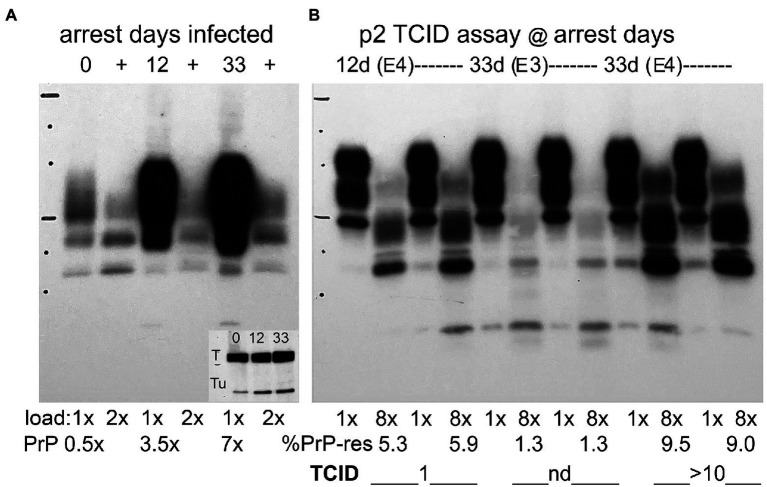
Panel **(A)** Representative Western blots of re-arrested infected FU-CJD cells at days indicated. Day 0 sample with parallel flasks arrested for 12 and 33 days. SEP cells show undigested PrP (E5 CE/lane) and adjacent PK+ lanes (+) have 2E5 CE/lane. PrP amounts increase to only 3.5 and 7x after 12 and 33 days of re-arrest. The % PrP-res (+ lanes) at day 0 is 44%, at day 12 is 7% and by day 33 decreases to 3%. The inset shows these re-arrested FU-CJD no longer inhibit Tag (T) unlike all other arrested cells. Tu is tubulin for load comparison. The inset in this blot shows tubulin (Tu) at days 0, 12 and 33 and confirms the lower cell load used for PrP quantitation. Panel **(B)** Assay of infectious titers at 12 and 33 days after arrest by quantitative *de novo PrP production* as described (Miyazawa et al., 2012). As early as p2, exposure to E4 re-arrested cells already show a TCID/cell of 1 at 12 days and this increases to and 10TCID/cell by 33 days; Cells exposed to 10-fold fewer re-arrested cells (E3) display low *de novo* PrP-res with a value not yet in the linear range for TCID quantitation (Liu et al., 2008). PK+ lanes are loaded at 8x the undigested lanes. %PrP-res is show below the blot with corresponding TCID/cell. Markers 25 kDa and 75 kDa are indicated by horizontal lines.

Figure 4A shows PrP and PrP-res (+ lanes) in a western blot of re-arrested FU-CJD cells at days 0, 12, and 33. The inset shows the Tag protein band in re-arrested FU-CJD at 12 and 33 days after re-arrest. No other arrested samples displayed this Tag band. Interestingly, Tag mRNA was not significantly increased at these days, making it is likely that the infectious agent itself, or other factors that it induces, stimulated Tag protein production (see section Discussion). PrP in these re-arrested cells, as in the arrested proliferating FU-CJD cells, was also comparatively reduced, yielding a 3.5x increase in PrP at 12 days, and only 7x by 33 days, again much lower than the ~30x levels of uninfected cells. The %PrP-res in these samples also progressively decreased during arrest. The highest %PrP-res is seen on day 0 (44%) while after re-arrest it declined by day 12 to 7% and by day 33 to only 3%.

Despite declining % PrP-res, the tissue culture infectious dose (TCID) increased significantly with re-arrest. Proliferating FU-CJD cells contained only 0.4 TCID/cell, whereas re-arrested cells showed 1 TCID/cell at 12 days, and by 33 days this increased 10 fold to 10 TCID/cell, i.e., an increase in infectivity of 25 fold. Figure 4, panel **B**, shows the TCID infectivity assay at 12 days and 33 days in re-arrested FU-CJD cells. *De novo* PrP-res was elicited only by infectious SEP samples in GT1 indicator cells, as previously described (Miyazawa et al., 2012), whereas infected cell homogenates display truncated PrP-res bands in the + lanes. As early as p2 after applying E4 cells from the 12 and 33 day samples, there is clear evidence of elicited *de novo* PrP-res at ≥5% in both samples; in these assays had values of 4–20% are in the quantitative TCID range of this assay. The duplicate re-arrested assay samples at p12 show a lower percent of *de novo* PrP-res (5.3 and 5.9% lanes) than at 33 days (9.5 and 9%), and re-arrested day 33 SEP cells at a 10-fold dilution (E3) do not yet show a PrP-response in the linear range (nd duplicate lanes with 1.3% PrP-res in each lane). Animal assays of infected cells have previously shown this TCID assay yields essentially the same infectivity levels as standard animal assays (Arjona et al., 2004). The high infectivity in SEP re-arrested cells at 33 days showed a low %PrP-res that did not predict the high titer of agent assayed. In fact, %PrP-res can be as high as 55% in both FU-CJD and kuru infections of neural GT1 mouse neuronal cells, yet kuru contains 1/100th the titer of FU-CJD in these cells (Miyazawa et al., 2011). More importantly, the huge IFN-β responses in re-arrested FU-CJD cells could not be attributed to PrP-res; FU-CJD proliferating and arrested cells both displayed a higher %PrP-res than re-arrested cells, yet both failed to increase IFN-β mRNA (see Figure 2 panel **C** and Figure 3 panel **C**).

## Discussion

The above data highlight three key changes induced by high titers of FU-CJD agent in re-arrested neuronal SEP cells: (1) enormous elevations of IFN-β RNA, (2) significantly diminished transcription of host PrP mRNA and protein production, and (3) the reappearance of Tag protein in re-arrested highly infectious cells. Unlike the PrP changes seen in both mRNA and protein, Tag protein reappeared only in re-arrested cells when Tag mRNA was low, underscoring the power of parallel RNA and protein measurements for identifying transcriptional versus translational controls. Delineation of sequential responses in neuronal lineage cells additionally uncovered major differences in immediate versus sustained molecular responses.

Unlike infected brain tissue which represents a diverse ecosystem of cell types that generally display only 2–4-fold mRNA changes (e.g., Kanata et al., 2019), infected neuronal SEP cells showed strikingly large changes in PrP and IFN-β mRNAs. Although prior analyses of purified infected microglia did not show any changes in PrP or IFN-β RNAs, they did show large elevations in innate immune inflammatory transcripts. These changes are biologically meaningful because they were detected in FU-CJD infected brains as early as 10–30 days after infection (Lu et al., 2004). Uninfected carrier brain did not show these changes. These previously characterized isolated microglial and whole brain responses underscore host recognition of the FU-CJD agent as a foreign entity. The huge IFN-β mRNA elevations induced by the FU-CJD agent in neuronal SEP cells is also typical for a host innate response to a foreign pathogen, one that is known to create a broad antiviral state involving many genes (Nagarajan, 2011; An et al., 2019).

Cell type comparisons are valuable for dissecting the role of different specialized cells in pathogenesis. The IFN-β mRNA elevations, as well as the PrP mRNA and protein reductions, may be specific for neuronal lineage cells because they are not found in microglial cells (Baker et al., 2004). The widespread, persistent human polyoma BKV virus can remain asymptomatic for many years, and it induces distinct interferon-related responses in different cell types that were related to persistence (An et al., 2019). In FU-CJD, different response patterns between long-lived non-proliferating microglia and re-arrested neuronal cells indicate distinct cellular roles in persistence and disease progression. Even with high RNA inputs for reverse transcription, IFN-β mRNA was undetectable in isolated brain microglia that contained high levels of FU-CJD infectivity by animal assay (Baker et al., 2004). For reference, FU-CJD infected mouse brain contains ~1 LD_50_/cell, equivalent to the infectivity of microglia (1 TCID/cell). Although highly infectious microglia contained barely detectable levels of host PrP, and no detectable misfolded PrP-res even at high gel loads, they mounted a complex transcriptional response to infection. This included >10-fold levels of interferon-responsive and innate immune pathway RNAs, such as CD72, when measured against noninfected non-migratory microglia (Baker et al., 2004). These innate immune RNA changes were not reproduced by misfolded PrP-res even when PrP-res was applied in large amounts to uninfected microglia. The inflammatory and interferon stimulatory pathways induced in microglia by the FU-CJD agent without an IFN-β RNA response indicate microglia, as long-lived myeloid dendritic cells, can recognize but not fully respond to a foreign TSE infectious particle. This is consistent with their role as reservoirs for agent persistence, one that can span >35 years in asymptomatic humans (Croes et al., 2002). The low PrP content of long-lived microglia and dendritic myeloid cells, in addition to their phenotypic characteristics, may also make them unable to support productive agent replication. In contrast, re-arrested neuronal SEP cells produced 25-fold increases in the FU-CJD agent in 33 days, i.e., 10x the infectivity/cell of brain. Clearly neurons bear the brunt of productive infection.

One other group has investigated IFN pathways using neural lineage cells: murine neuroblastoma N2a-58 cells persistently infected with 22 L scrapie (Ishibashi et al., 2019). These proliferating cells were never tested for neural differentiation or sustained effects, unlike SEP cells above, and their infectivity was not reported. N2a-58 cells infected with the 22 L scrapie agent in our lab have been biologically heterogeneous, as would be expected for malignant tumor cells, and single cell subclones yielded relatively low and variable infectious 22 L scrapie titers ranging from only 0.1 to <0.001 TCID/cell (Liu et al., 2008). In contrast, naive, newly infected SEP cells (Miyazawa et al., 2012) as well as re-arrested FU-CJD SEP cells above reproducibly contain 1–10 TCID/cell, considerably greater than our 22 L scrapie N2a-58 cells. Such differences between SEP cells and 22 L scrapie N2a-58 neuroblastoma cells may explain why IFN-β RNA expression was so different in our experiments. For example, other’s infected N2a-58 cells showed a small 2-fold *decrease* in IFN-β RNA (Ishibashi et al., 2019), in sharp contrast to the sustained 40-68x-fold increase in IFN-β RNA signals in high-titer re-arrested SEP cells. Those authors also concluded that “infectious prions, i.e., PK resistant PrP^sc^ (PrP-res), cause the suppression of endogenous interferon expression.” However, recombinant IFN-β applied to their 22 l scrapie N2a-58 cells reduced PrP-res by 50% at 48 h, suggesting that the broad transcriptional effects of IFN-β peptide reduced PrP-res, not vice versa. In SEP cells the 68-fold increase in IFN-β mRNA was provoked only by the highest titer 33-day re-arrested cells, not low titer persistently infected cells, and FU-CJD proliferating and arrested cells with low infectivity, but visible PrP-res bands, produced no change in IFN-β mRNA. Finally, a lower 8-fold increase in IFN-β RNA, not related to the initial, possibly stressful shift in temperature/serum used to induce arrest, was sustained in uninfected differentiated SEP cells. This raises the possibility that this cytokine may participate in neural differentiation.

In contrast to IFN-β mRNA elevations, FU-CJD inhibited the PrP mRNA changes induced by arrest. Whereas uninfected SEP cells stably produced 30-fold increases in PrP mRNA when arrested at passages 7 through 25, the arrested and re-arrested p17 FU-CJD SEP cells produced only 2.5–5.4-fold PrP mRNA elevations, respectively. Uninfected cells also sustained high levels of PrP mRNA for 26 days whereas the arrested and re-arrested FU-CJD cells showed only a transient early increase in PrP mRNA with a rapid decline to ≤2 fold. Protein synthesis was also sharply decreased. This PrP reduction might limit susceptibility to further infection. However, these reductions in PrP mRNA and protein were insufficient to inhibit substantial agent replication as demonstrated in infectivity assays. The presence of PrP-res in FU-CJD proliferating cells also had no effect on PrP mRNA and displayed the same 1x level as uninfected proliferating cells without PrP-res. A quite different host anti-FU-CJD agent response was observed previously in arrested FU-CJD infected cells maintained for 200 days. These SEP cells accumulated huge amounts of PrP-res, and eventually eliminated all five logs of infectivity (Miyazawa et al., 2012). This accumulated old PrP-res was not infectious. Instead, these PrP-res amyloid fibrils can trap infectious agent particles and eventually eliminate them as part of a final host defence.

The third key finding was the reappearance of Tag in FU-CJD re-arrested cells with high infectivity. This proliferative effect was seen at the protein, not transcriptional level. SEP cells infected with CJD have previously shown increased proliferation at later passages when compared with uninfected controls (Miyazawa et al., 2012). CJD infection can lead to increased cellular proliferation in cell lines with transformation effects sufficient to produce large tumors in animals (Manuelidis et al., 1987, 1988). This data again suggests that TSE agents can turn on cellular proliferative genes, as do persistent polyoma DNA viral infections (An et al., 2019). Cellular proliferation also critically changes the effective doubling times (*t_i_*) of TSE agents; in wild type mice agent doubling times in brain are prolonged, and different strains show doubling times that range from 6.7 to 33 days (Miyazawa et al., 2011). Re-arrested SEP cells showed a 25-fold increase in FU-CJD agent titer over 33 days, yielding an agent *t_i_* doubling time of 4.6 days, shorter than the *t_i_* of 6.7 days of FU-CJD agent in mouse brain, but still prolonged. In contrast, when FU-CJD and other TSE agents are transferred to dividing GT1 neuronal cells they all show a *t_i_* of only 1 day, and this parallels the doubling time of their host GT1 cells (Miyazawa et al., 2011). TSE agent doubling times are therefore intimately linked to cellular DNA proliferation and its polymerases. Finally, IFN-β induces a cascade of transcriptional and other innate immune response genes that are virucidal for RNA viruses, yet DNA viruses, such as human polyoma BKV, can induce an antiviral interferon state yet continue to persist (An et al., 2019). This points to a DNA rather than an RNA as the nucleic acid essential for TSE particle infection. Indeed, highly infectious particle preparations have negligible RNA but contain a variety of DNAs including circular DNAs that have an environmental origin (Manuelidis, 2011). When these DNAs are destroyed, infectious particles lose ~3 logs of infectivity.

RNA-seq can give a far more complete picture of the pathways involved in agent-host interactions in FU-CJD infected SEP cells and illuminate specific pathways that induce IFN-β mRNA elevations. A virus induced reduction of its own targeted viral attachment molecule, as host PrP, by transcriptional silencing may be rare. Instead, PrP mRNA degradation may have a greater role in FU-CJD induced PrP reductions. Epigenetic RNA modifications can have strong effects on mRNA stability, and include chemical methylated base changes, modification inducers, destabilization enzymes and erasers (Boo and Kim, 2020), and viruses can manipulate mRNA decay through such changes (Narayanan and Makino, 2013). In progressive sCJD mouse brain infection, elevated RNA editing transcripts that can contribute to mRNA breakdown have been identified (Kanata et al., 2019).

Phase separation studies of viral components show they can interact with host proteins to hijack host cell functions (Saito et al., 2021) or inhibit immune recognition by the host (e.g., Heinrich et al., 2018). Host DNA sensors are cytosolic (as are TSE infectious particles) and can respond to viral DNA and induce IFN-β (Nagarajan, 2011). An FU-CJD DNA might be exposed during particle-PrP interactions to induce IFN-β. Phase separation studies can also facilitate identification of other proteins that interact with TSE particle nucleic acids to inhibit progressive agent replication and protection. In contrast, late stage aggregates of PrP amyloid, as other amyloids and fibrillary tangles, remain flags of a sinking ship that gives no clue about the initiating causes of later neurodegeneration. In TSEs, the initiating cause is clearly an infectious agent with viral characteristics. Given the evidence, interrogating virus-like particles for their associated molecules, including nucleic acids, will lead to a better understanding of TSE pathogenesis and complex agent-host interactions in different cell types. Such interrogations can reveal a broader group of molecular targets for effective therapy at latent non-symptomatic stages of disease.

## Data Availability Statement

The original contributions presented in the study are included in the article/Supplementary Material, and further inquiries can be directed to the corresponding author.

## Author Contributions

GA and NP performed the majority of RNA and RT/qPCR, protein blot, tissue culture, infectivity assays, and also contributed to data analysis. LM designed the project experiments, analyzed data, prepared data presentation, and wrote the manuscript. All authors contributed to the article and approved the submitted version.

## Funding

This work was supported by the William H. Prusoff Foundation.

## Conflict of Interest

The authors declare that the research was conducted in the absence of any commercial or financial relationships that could be construed as a potential conflict of interest.

## Publisher’s Note

All claims expressed in this article are solely those of the authors and do not necessarily represent those of their affiliated organizations, or those of the publisher, the editors and the reviewers. Any product that may be evaluated in this article, or claim that may be made by its manufacturer, is not guaranteed or endorsed by the publisher.

## Abbreviations

SEP: Post-mitotic rat septal neurons immortalized by large T antigen (Tag) from a simian polyoma virus (SV40)
CJD: Creutzfeldt-Jakob disease
BSE: Bovine spongiform encephalopathy
TSEs: Transmissible spongiform encephalopathies
sCJD: Sporadic CJD
FU-CJD: A CJD agent isolated in Japan
PrP: Host prion protein
PrP-res: PrP resistant to limited protease digestion
LD_50_: Lethal dose for 50% of inoculated animals
WBC: White blood cells
RT/qPCR: Method for quantifying mRNA transcripts
Cq: Quantification cycle by RT/qPCR
TCID: Tissue culture infectious dose per cell
RT: Reverse transcriptase
GAPDH: Glyceraldehyde-3-phosphate dehydrogenase
NFP: Neurofilament protein
IFN: Interferon
Tm: Melting temperature
HEK: Human embryonic kidney cells
OAS: 2′-5′ oligoadenylate synthetase
CE: Cell equivalents
t_i_: Effective doubling time of TSE agent strains
