# Supplementary material for: Reduced Expression of Prion Protein With Increased Interferon-β Fail to Limit Creutzfeldt-Jakob Disease Agent Replication in Differentiating Neuronal Cells
Source: Front Physiol. 2022 Feb 18;13:837662. doi: 10.3389/fphys.2022.837662 (PMC8895124; doi:10.3389/fphys.2022.837662)
Supplement: Supplementary file 1 [file Data_Sheet_1.docx]

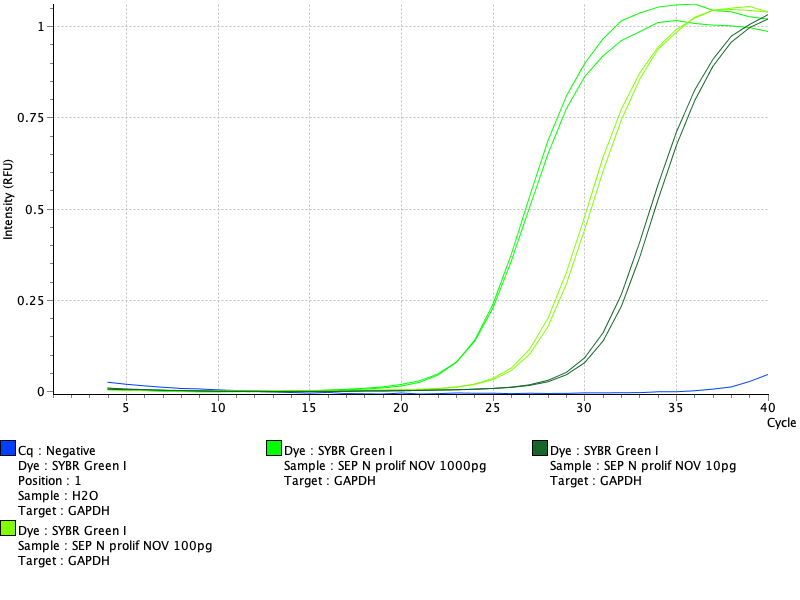


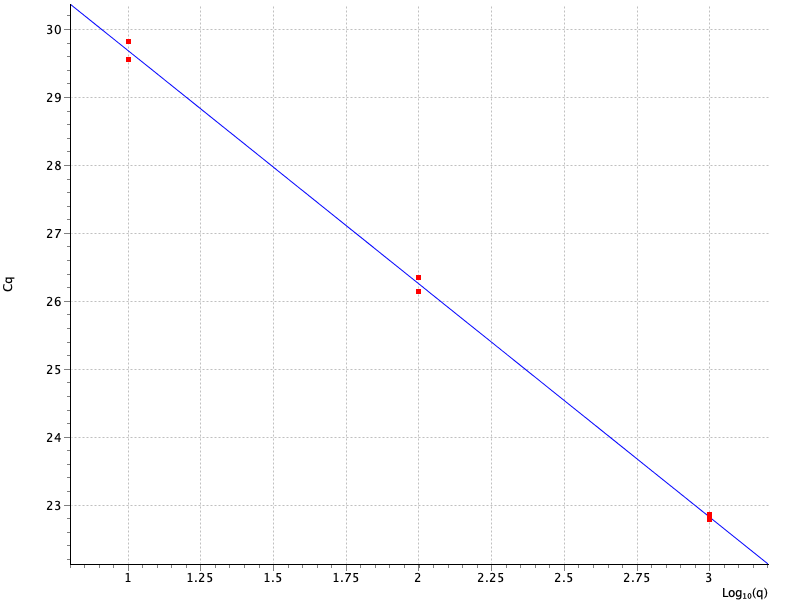


R^2^= 0.9987

y=-3.43x+33.11

**Supplement Fig 1**. Top panel: Example of GAPDH standard 10 fold dilution curves from uninfected proliferating SEP RNAs shown in top graph. Each duplicate GAPDH dilution is shown in three different green tones, along with a water blank control (blue) that shows no signal in this plot of fluorescent intensity (y) versus Cq (x). Bottom panel graph shows corresponding linear regression and R^2^ value from these serial 10 fold dilutions of RNA. Plot shows Cq (y) versus Log_10_ RNA amount (x) used to calculate GAPDH RNA in experimental samples. Total RNA input for these standard RT-qPCR dilutions were 1000pg, 100pg and 10pg.


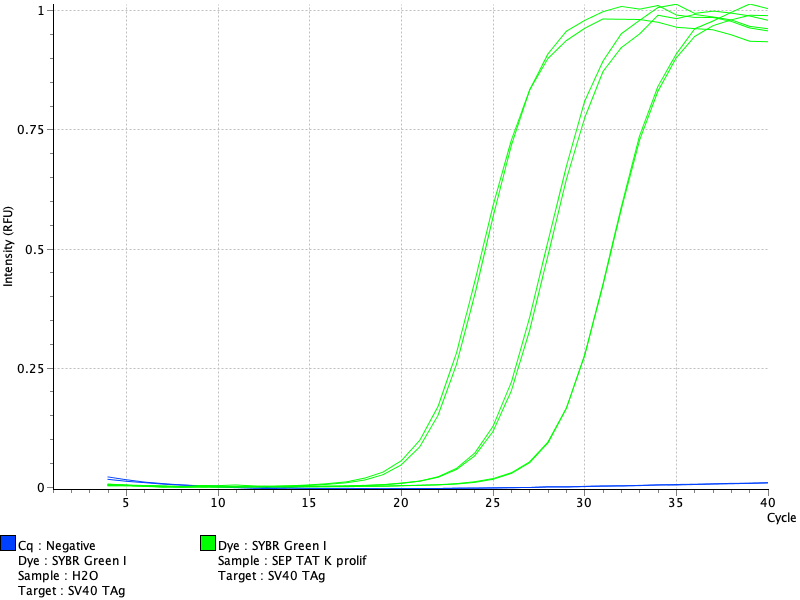

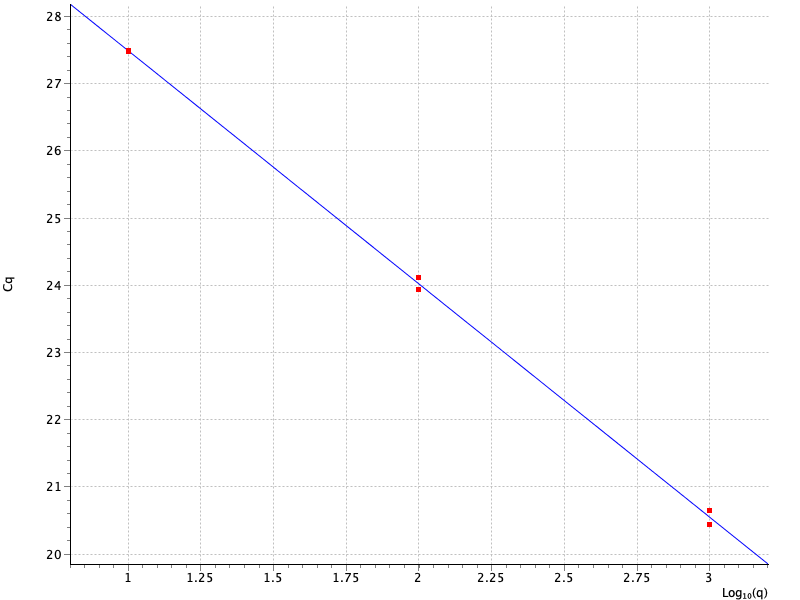


R^2^= 0.9993

y=-3.47x+30.95

**Supplement Fig 2**. Top panel: Example of SV-40 Tag standard 10 fold dilution curves (1000pg, 100pg and 10pg of RNA from a representative infected proliferating SEP sample with load normalized by GAPDH). Each sample dilution (green) was run along with a water blank control (blue) that shows no signal in this plot of fluorescent intensity (y) versus Cq (x). Bottom panel graph shows corresponding linear regression and R^2^ value from these serial 10 fold dilutions of RNA. Plot shows Cq (y) versus Log_10_ RNA amount (x). This same procedure was performed for every primer pair to ensure the RNA quantity loaded in the RT-PCRs reactions gave the Cq value linear range.


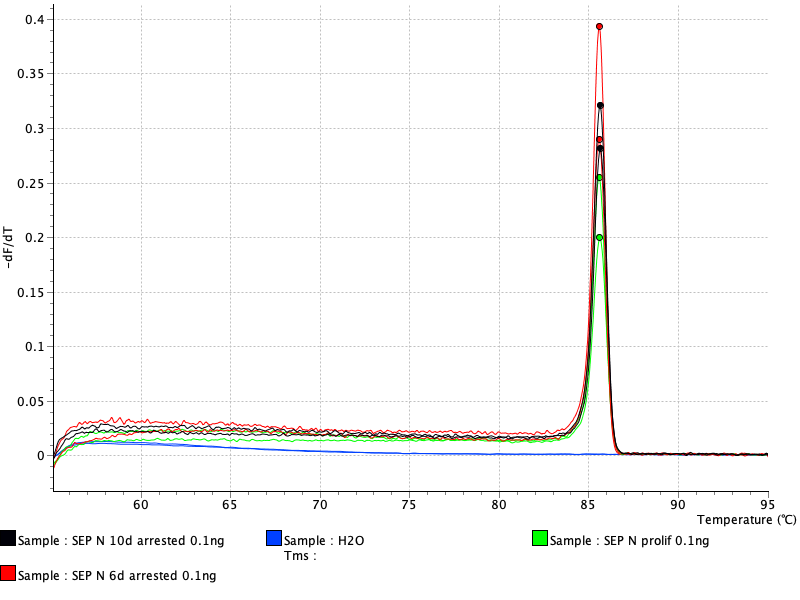


**Supplement Fig 3**. Example of PrP RT-qPCR melting peaks analysis shows no spurious peaks other than the single one of the RT-qPCR products. -dF/dT (y) versus temperature in ^o^C (x) was plotted, where -dF/dT is the derivative of the function fluorescence vs. temperature. Each sample (green for proliferating, red/black for arrested) was done in duplicate with two water blank controls (blue). Melting peaks were the same for all primer pairs in infected and uninfected cells.


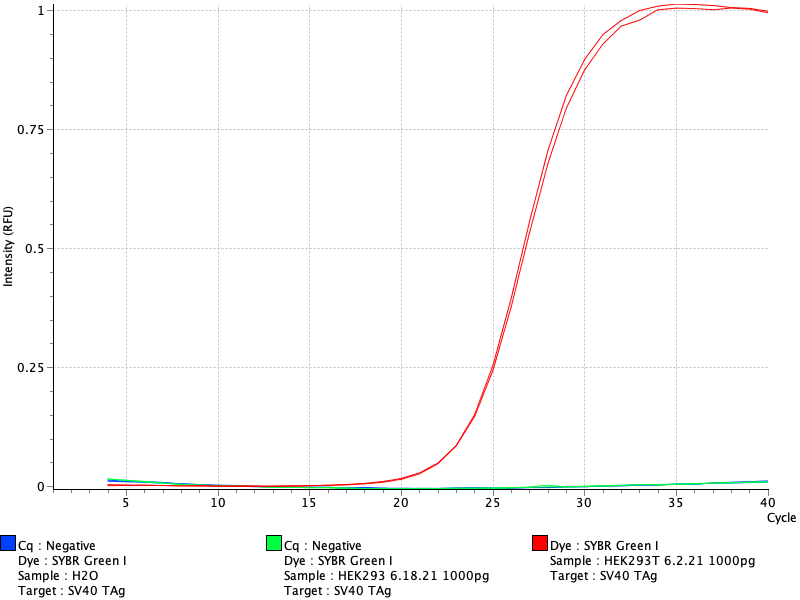


**Supplement Fig 4**. Fluorescence signal intensity (y) versus cycle number (x) is plotted. The samples are negative control HEK cells without Tag (green) and positive HEK Tag+ cells (red). Only Tag + cells show a positive signal for SV-40 Tag amplification. The RNA load for these reactions was 1000pg and a water blank control (blue) was run at the same time. Early passage HEK Tag - and Tag + cells were a gift of Ann Edwards and Daniel DiMaio.


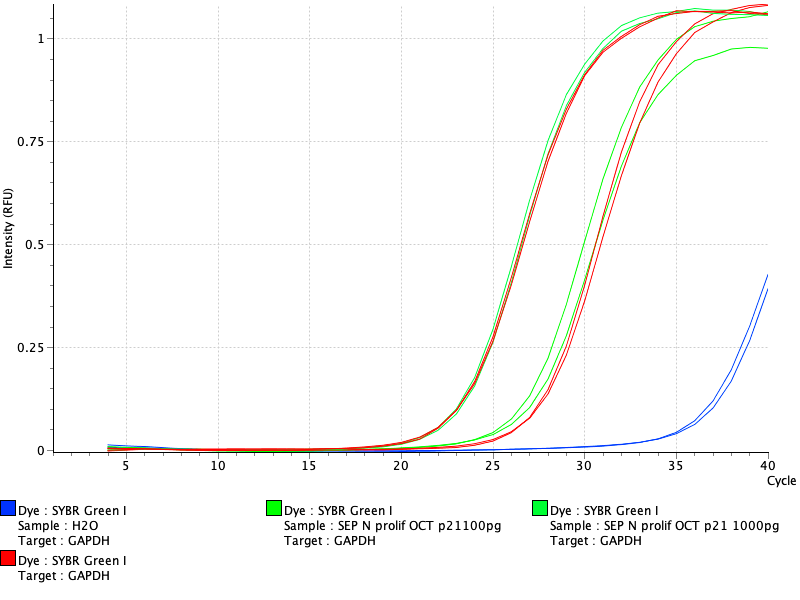


**Supplement Fig 5**. Fluorescence signal intensity (y) versus cycle number (x) is plotted. The samples are normal (green) and infected (red) SEP proliferating RNA. GAPDH RT/qPCR curves from both samples at 1000pg and 100pg showed comparable curves with no significant differences between them (Cq values were 26.1 and 26.8 respectively for 100pg input, 22.47 and 22.5 respectively for 1000pg input). A water blank control was run at the same time (blue).
